# BMI and HbA1c are metabolic markers for pancreatic cancer: Matched case-control study using a UK primary care database

**DOI:** 10.1371/journal.pone.0275369

**Published:** 2022-10-05

**Authors:** Agnieszka Lemanska, Claire A. Price, Nathan Jeffreys, Rachel Byford, Hajira Dambha-Miller, Xuejuan Fan, William Hinton, Sophie Otter, Rebecca Rice, Ali Stunt, Martin B. Whyte, Sara Faithfull, Simon de Lusignan

**Affiliations:** 1 Faculty of Health and Medical Sciences, University of Surrey, Guildford, United Kingdom; 2 Royal Surrey NHS Foundation Trust, Guildford, United Kingdom; 3 Nuffield Department of Primary Care Health Sciences, University of Oxford, Oxford, United Kingdom; 4 Primary Care Research Centre, University of Southampton, Southampton, United Kingdom; 5 Barnardo’s, Barkingside, Ilford, Essex, London, United Kingdom; 6 Pancreatic Cancer Action, London, United Kingdom; The University of Mississippi Medical Center, UNITED STATES

## Abstract

**Background:**

Weight loss, hyperglycaemia and diabetes are known features of pancreatic cancer. We quantified the timing and the amount of changes in body mass index (BMI) and glycated haemoglobin (HbA1c), and their association with pancreatic cancer from five years before diagnosis.

**Methods:**

A matched case-control study was undertaken within 590 primary care practices in England, United Kingdom. 8,777 patients diagnosed with pancreatic cancer (cases) between 1^st^ January 2007 and 31^st^ August 2020 were matched to 34,979 controls by age, gender and diabetes. Longitudinal trends in BMI and HbA1c were visualised. Odds ratios adjusted for demographic and lifestyle factors (aOR) and 95% confidence intervals (CI) were calculated with conditional logistic regression. Subgroup analyses were undertaken according to the diabetes status.

**Results:**

Changes in BMI and HbA1c observed for cases on longitudinal plots started one and two years (respectively) before diagnosis. In the year before diagnosis, a 1 kg/m^2^ decrease in BMI between cases and controls was associated with aOR for pancreatic cancer of 1.05 (95% CI 1.05 to 1.06), and a 1 mmol/mol increase in HbA1c was associated with aOR of 1.06 (1.06 to 1.07). ORs remained statistically significant (*p* < 0.001) for 2 years before pancreatic cancer diagnosis for BMI and 3 years for HbA1c. Subgroup analysis revealed that the decrease in BMI was associated with a higher pancreatic cancer risk for people with diabetes than for people without (aORs 1.08, 1.06 to 1.09 versus 1.04, 1.03 to 1.05), but the increase in HbA1c was associated with a higher risk for people without diabetes than for people with diabetes (aORs 1.09, 1.07 to 1.11 versus 1.04, 1.03 to 1.04).

**Conclusions:**

The statistically significant changes in weight and glycaemic control started three years before pancreatic cancer diagnosis but varied according to the diabetes status. The information from this study could be used to detect pancreatic cancer earlier than is currently achieved. However, regular BMI and HbA1c measurements are required to facilitate future research and implementation in clinical practice.

## Introduction

Pancreatic cancer is a devastating disease. It is the tenth most common cancer in the United Kingdom and 14^th^ globally accounting for around 3% of all new cancer cases [1]. Each year there are over 10,000 new cases in the United Kingdom [2], over 60,000 in the United States and nearly half a million worldwide [1]. Unfortunately, the survival statistics are very poor for pancreatic cancer compared to other cancers. The median survival is nine months and less than 10% of people survive five years or more after diagnosis [3, 4]. The high mortality rate of pancreatic cancer is attributed to late diagnosis. Over 80% of people are diagnosed at the advanced, lethal stages when the cancer has spread outside the pancreas to the liver or other organs. One way to improve early diagnosis is by learning more about pancreatic cancer symptoms, quantifying the risk, and by learning how to identify people at an increased risk. Older age, diabetes mellitus, chronic pancreatitis, and gallbladder disease are established risk factors [5]. Symptoms include weight loss (often severe), increasing blood glucose levels (often rapid), back pain, abdominal pain and gastrointestinal problems [6, 7]. Because these symptoms are unspecific, pancreatic cancer diagnosis remains challenging. The key UK guidelines on pancreatic cancer diagnosis and treatment by the National Institute for Health and Care Excellence (NICE) do not include weight loss or loss of glycaemic control as diagnostic features [8]. However, the general cancer guidelines by NICE do recommend an urgent abdominal scan in people over 60 with weight loss and new-onset diabetes or other symptoms [9].

Prediction algorithms have been developed that in the future could find clinical applications in improving early diagnosis and in population-based screening. Some, such as the Enriching New-Onset Diabetes for Pancreatic Cancer (ENDPAC) algorithm are based on age, weight, and glucose [10, 11]. However, they are validated only for people with new onset of diabetes. Other algorithms are more complex and use machine learning and an algorithm-driven selection of symptoms and risk factors [7, 12, 13], but these require large amounts of good quality symptom data. In clinical practice, pancreatic cancer is relatively rare. It is estimated that a primary care clinician will only see three to four cases in their working lifetime. Therefore, data-based approaches are required to support clinicians in identifying patients with an increased risk.

We designed this case-control study to show longitudinal trends and quantify the association of body mass index (BMI) and glycated haemoglobin (HbA1c) with pancreatic cancer from five years before diagnosis. We described the pancreatic cancer cohort to analyse how many cases received diabetes diagnosis and when in relation to pancreatic cancer. We visualised and compared changes over time in BMI and HbA1c between cases and controls to show how early changes in BMI and HbA1c develop before pancreatic cancer diagnosis and by how much. We calculated odds ratios over time for BMI and HbA1c to quantify the risk of pancreatic cancer. This evidence could be used to improve understanding of symptoms trajectory in pancreatic cancer, which patients are at an increased risk of pancreatic cancer, and when clinicians should be most vigilant. Research to date focuses on estimating the risk of pancreatic cancer in people with new diabetes. However, because only 40% to 50% of people with pancreatic cancer will develop diabetes [14, 15], we undertook subgroup analysis according to the diabetes status. This was to compare changes in BMI and HbA1c between cases with and without diabetes.

In its advanced stage, pancreatic cancer progresses rapidly, and patients generally do not respond to curative treatment. There is evidence that pancreatic cancer takes many years (potentially up to 20 years) to develop and exhibit metastatic activity [16]. Therefore, if an improved understanding of risk factors could help clinicians detect pancreatic cancer months or even years earlier than currently, this could expedite life-saving surgery.

## Material and methods

### Dataset

The Oxford-Royal College of General Practitioners Clinical Informatics Digital Hub (ORCHID) database was used. ORCHID is a nationally representative database [17]. It downloads electronic healthcare records (EHRs) from primary care providers (GP practices) that belong to the sentinel network of the Royal College of General Practitioners, Research and Surveillance Centre [18]. The network comprises 1,800 practices serving more than 15 million people in England and Wales, United Kingdom. In October 2020, a dataset was extracted for this study using the systematized nomenclature of medicine clinical terminology (SNOMED CT) system applying previously described extraction approaches [19, 20]. The list of SNOMED CT concepts can be obtained from the corresponding author. The dataset included information on demographics, pancreatic cancer diagnosis, diabetes, and pancreatic cancer features (abdominal pain, back pain, change in bowel habits, constipation, diarrhoea, nausea, vomiting, abdomen scan, operation on pancreas, jaundice, weight loss, suspected pancreatic cancer, pancreatic cancer referral) as well as all BMI and HbA1c measurements recorded in 2001 or after.

### Study design

This was a matched case-control study. Practice inclusion was limited to practices passing data quality control. Participants were included in the matching process if they either had a pancreatic cancer diagnosis, or any of the listed above pancreatic cancer features at the age of ≥ 40 years. The study sample included 3,539,397 adults registered within 590 practices.

#### Cases

We identified 8,811 EHRs of people with incident pancreatic cancer diagnosed at ≥ 18 years in 2007 or after. Pancreatic cancer diagnosis, defined as the first time that a SNOMED CT code for pancreatic cancer was recorded in an EHR, was an index date. Any malignant forms of primary pancreatic cancer were included. Benign, in-situ and secondary cancers were excluded. We used the 2007 cut-off because coding of the metabolic markers such BMI and HbA1c in primary care improved significantly from 2004 with the introduction of the Quality and Outcomes Framework and the pay-for-performance incentive scheme [20]. Therefore the 2007 cut-off was set to improve data completeness for at least 3 years before the index date.

#### Controls

Controls were matched to cases by age (year of birth), gender, and the type and duration of diabetes (if present). A maximum of four randomly selected EHRs without pancreatic cancer diagnosis from a pool of available matches within the study sample were selected. Controls did not have a pancreatic cancer diagnosis, so their index date was the date of pancreatic cancer diagnosis for their respective cases. The median number of available matched controls per case was 14,324 (range 0 to 35,421). 34 cases were excluded because they did not have a matched control, and this was due to the coding of diabetes. Specifically, 30 cases without controls had type 1 diabetes recorded at the median age of 71. The remaining 4 cases for which controls could not be found, had type 2 diabetes recorded at the ages between 11 and 38. The matching process resulted in the final study population of 8,777 cases and 34,979 controls.

### Data preparation

BMI and HbA1c are both challenging types of data to model statistically. They are opportunistically recorded during healthcare processes, so they are often incomplete and recorded at irregular intervals. We extracted all available BMI and HbA1c data for cases and controls from six years before the index date and up to a year after. We analysed these data in the following three ways:

1. To visualise the trends in BMI and HbA1c in plots over time, we calculated raw monthly averages. To smooth the raw data, all available data were fitted with linear regression. Changes in BMI and HbA1c were modelled as a function of time with a three-knot cubic spline to allow nonlinearity in trends over time.
2. For the table that summarises population characteristics at the time of pancreatic cancer diagnosis, BMI and HbA1c values nearest to the index date were used for each participant from the period of ±1 year. Where there was no BMI or HbA1c measurement in that time window, data were reported as missing.
3. And finally, for the conditional logistic regression, to allow modelling over time, a data table was constructed with average BMI and HbA1c values per person per year starting from year -5 before the index date. This was to account for multiple BMI and HbA1c measures recorded for many participants across the year. For example, for the year -1 model, measurements were averaged for each participant from the time window of -365 days up to the index date. For the year -2 model, average BMI and HbA1c values were calculated for each person from the time window between -730 days to -366 days. For the year 0 model, measurements were averaged from the time window between the index date and 365 days after index date. The average was chosen as a summary statistic for regression analysis, to account for irregularity in timing of data collection. For participants who did not have at least one BMI and HbA1c measurement, the data in that year were set to missing.

To undertake regression modelling, the data table that included BMI and HbA1c from 6 points in time, demographic information, all covariates as well as the cancer diagnosis variable (the case-control binary index) was treated with multiple imputation for missing data as explained below, and modelled with conditional logistic regression. The results are reported based on complete cases and from the multiple imputed datasets combined in accordance with the Rubin’s rule [21].

### Statistical analysis

To describe the demographic information for the study population, descriptive statistics were used such as means with 95% confidence intervals (CI), medians with interquartile ranges (IQR), and counts with percentages. The proportion of missing data was also described using counts with percentages. We assumed that data were missing at random, so means with CIs and counts with percentages, in the population summary table, were calculated based on available data (the sample for which data were recorded). Multiple imputation by chained equations with fifty imputations was used to account for a large proportion of missing data [21].

BMI and HbA1c were plotted over time for cases and controls from 6 years prior pancreatic cancer diagnosis (index date for controls). Subgroup analyses were undertaken according to gender and the diabetes status (separately for people with and without diabetes).

Odds ratios (OR) of pancreatic cancer for differences in BMI and HbA1c between cases and controls were calculated with conditional logistic regression to account for matching in the study population by age, gender and diabetes. Regression models were calculated for the total population, and in two subgroups by diabetes status (people with diabetes and people without diabetes), at six points in time, starting from five years before the diagnosis (year -5 model, year -4, year -3, year -2, year -1, year 0). Adjusted models included BMI and HbA1c measurements for that year, ethnicity, index of multiple deprivation (IMD) as quintiles, smoking and alcohol consumption. They did not include BMI and HbA1c measurements from previous years. This is because BMI and HbA1c variables from subsequent years were highly correlated with each other and including them in one model would cause multicollinearity. Multicollinearity was assessed with the variance inflation factors (VIF) analysis and VIF > 10 as the threshold that indicated significant multicollinearity [22]. All the variables included in the model had VIF values < 1.1 indicating no significant collinearity among them.

To account for multiple comparisons, the statistical significance was set at *p* < 0.01 but 95% CIs were provided to facilitate comparisons with other published studies. The database was managed in Structured Query Language (SQL) Server Management Studio version v18.9.1 and analyses were undertaken with R Core Team (2021) software version 4.1.0 using available R functions such as mice and clogit. The STROBE guidelines (Strengthening the Reporting of Observational Studies in Epidemiology) were followed [23].

## Results

### Participants

**Fig 1:**
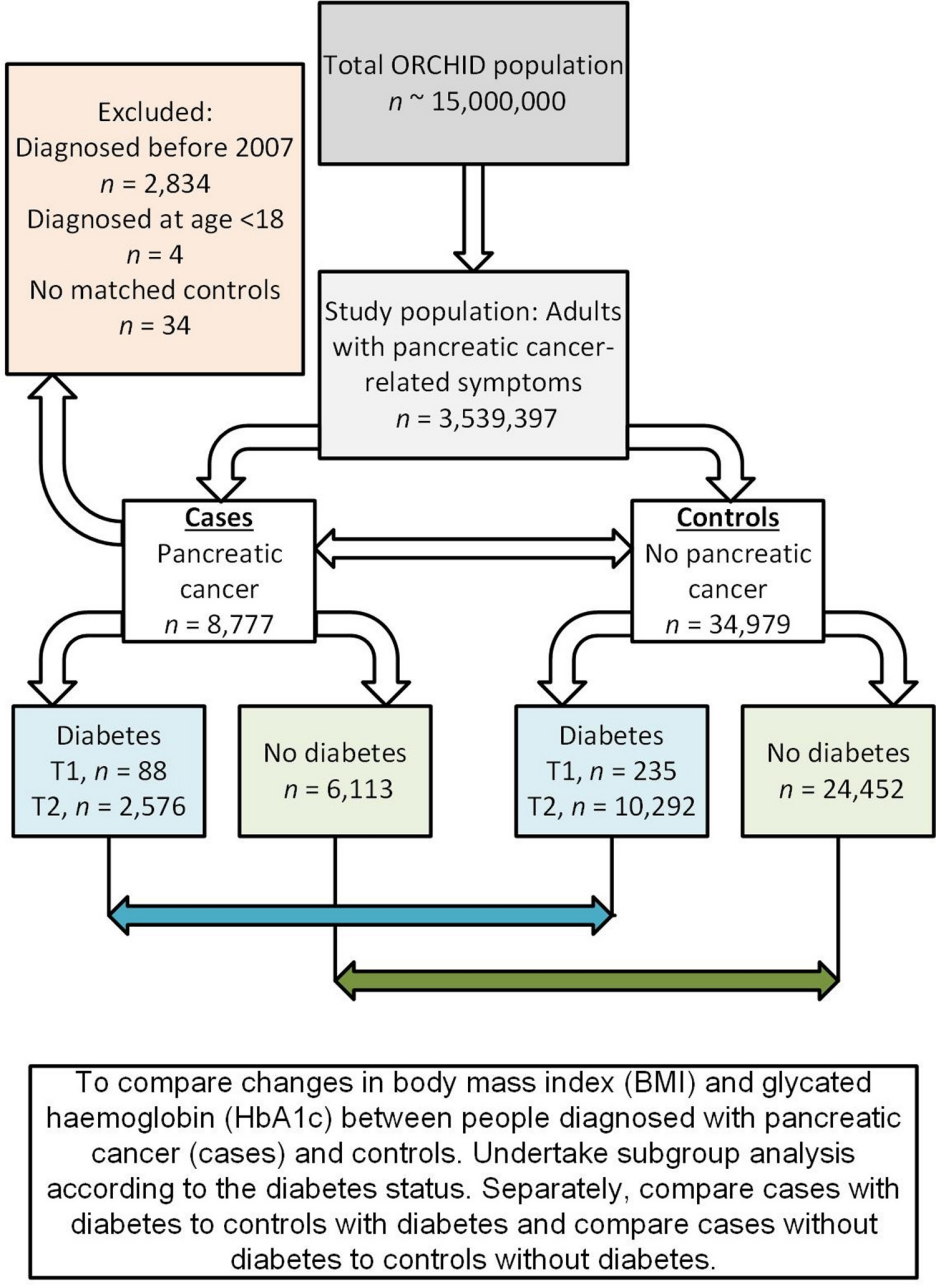
Study participants, a consort diagram illustrating the construction of the case-control dataset and plan of the analysis to fulfil the aims and objectives of this study.

The study population included 8,777 cases diagnosed with pancreatic cancer between 1^st^ January 2007 and 31^st^ August 2020 and 34,979 matched controls with an even 50%:50% split between males and females. The consort diagram in Fig 1 depicts the construction of the case-control study population and Table 1 provides detailed characteristics of the total study population and by diabetes subgroups. Only 30% of people with pancreatic cancer received diagnosis of diabetes, and this was mostly in the same year or after pancreatic cancer diagnosis. More specifically, there were 88 (1%) cases with type 1 diabetes and 2,576 (29%) with type 2 diabetes, 52 (59%), and 663 (26%) of these respectively, received diabetes diagnosis in the same year or after pancreatic cancer diagnosis.

**Table 1:** Characteristics of the study sample. Comparison of pancreatic cancer cases and controls using crude (unadjusted) odds ratios (OR) and 95% confidence intervals (CI) calculated with conditional logistic regression to account for matching on age, gender and diabetes. Subgroup analysis of body mass index (BMI) and glycated haemoglobin (HbA1c) at diagnosis were presented by diabetes status. Means (95% CIs), medians (interquartile ranges [IQR]), and counts (%) were calculated based on available data.

	Cases (*n* = 8,777) *n* (%) or mean (95% CI)	Controls (*n* = 34,979) *n* (%) or mean (95% CI)	Crude OR (95% CI)	*p*-value
Age at index date (years)	72.1 (71.9 to 72.3)	72.1 (72.0 to 72.2)	Matched	
Median (IQR) (years)	73 (65 to 81)	73 (65 to 81)	-	-
Gender			Matched	
Female	4,366 (49.7)	17,416 (49.8)	-	-
Male	4,411 (50.3)	17,563 (50.2)	-	-
Ethnicity				
White	5,853 (94.4)	22,837 (92.4)	Reference	
Asian	178 (2.9)	1,099 (4.5)	0.6 (0.5 to 0.7)	<0.001
Black	115 (1.9)	548 (2.2)	0.8 (0.7 to 1.0)	0.069
Mixed	27 (0.4)	105 (0.4)	1.1 (0.7 to 1.7)	0.691
Other	27 (0.4)	132 (0.5)	0.7 (0.5 to 1.1)	0.158
Missing	2,577 (29.4)	10,258 (29.3)	-	-
Index of multiple deprivation (IMD) score				
Quintile 5 (least deprived)	2,162 (25.4)	8,339 (24.7)	Reference	
Quintile 4	2,050 (24.1)	7,652 (23.1)	1.0 (0.9 to 1.1)	0.879
Quintile 3	1,866 (22.0)	7,106 (20.8)	1.0 (0.9 to 1.1)	0.656
Quintile 2	1,348 (15.9)	5,799 (17.0)	0.9 (0.8 to 1.0)	0.011
Quintile 1	1,071 (12.6)	4,875 (14.4)	0.8 (0.8 to 0.9)	<0.001
Missing	280 (3.2)	1,208 (3.5)	-	-
Smoking status				
Non-smoker	1695 (24.6)	7091 (25.3)	Reference	-
Active smoker	1110 (16.1)	3371 (12.1)	1.4 (1.3 to 1.6)	<0.001
Ex-smoker	4076 (59.2)	17602 (62.7)	1.0 (0.9 to 1.0)	0.356
Missing	1896 (21.6)	6915 (19.8)	-	-
Harmful alcohol usage				
No	8,648 (98.5)	34,439 (98.5)	Reference)	-
Yes	129 (1.5)	540 (1.5)	0.9 (0.8 to 1.2)	0.633
Diabetes: type 1			Matched	
Yes	88 (1.0)	235 (0.7)	-	-
No	8,689 (99.0)	34,744 (99.3)	-	-
Age at diabetes diagnosis (years)	56.0 (52.2 to 59.8)	53.4 (51.3 to 55.4)	-	-
Median age (IQR) (years)	60.0 (47.0 to 68.0)	56.0 (46.0 to 64.5)	-	-
Median duration of diabetes at the index date (IQR) (years)	0.0 (0.0 to 7.0)	0.0 (0.0 to 8.5)	-	-
Diabetes diagnosed from ≥5 to ≤1 years before the index date	12 (13.6)	40 (17.0)	-	-
Diabetes diagnosed ≥0 years before the index date	52 (59.1)	121 (51.5))	-	-
Diabetes: type 2			Matched	
Yes	2,576 (29.3)	10,292 (29.4)	-	-
No	6,201 (70.7)	24,687 (70.6)	-	-
Age at diabetes diagnosis (years)	67.2 (66.7 to 67.6)	67.2 (67.0 to 67.4)	-	-
Median age (IQR) (years)	68.0 (60.0 to 75.0)	68.0 (60.0 to 75.0)	-	-
Median duration of diabetes at the index date (IQR) (years)	4.0 (0.0 to 10.0)	4.0 (0.0 to 10.0)	-	-
Diabetes diagnosed from ≥5 to ≤1 years before the index date	831 (32.3)	3,324 (32.3)	-	-
Diabetes diagnosed ≥0 years before the index date	663 (25.7)	2,652 (25.8)	-	-
BMI at index date (±1 year, the nearest recorded value) (kg/m^2^)	25.7 (25.6 to 25.8)	28.4 (28.3 to 28.5)	1.6 (1.5 to 1.7) for decrease (weight loss) by 5 units	<0.001
Missing	3,271 (37.3)	19,181 (54.8)	-	-
BMI class				
Underweight (<18.5)	313 (5.7)	342 (2.2)	1.6 (1.3 to 2.0)	<0.001
Normal (≥18.5 to <25)	2424 (44.0)	4265 (27.0)	Reference	-
Overweight (≥25 to <30)	1775 (32.2)	5929 (37.5)	0.5 (0.5 to 0.6)	<0.001
Obese (≥30)	994 (18.1)	5262 (33.3)	0.3 (0.3 to 0.4)	<0.001
HbA1c at index date (±1 year, the nearest recorded value) (mmol/mol)	55.0 (54.4 to 55.7)	48.5 (48.2 to 48.7)	1.4 (1.4 to 1.5) for increase by 10 units)	<0.001
Missing	4383 (49.9)	22,577 (64.5)	-	-
**Subgroup analysis–people with diabetes**	***n* = 2,664**	***n* = 10,527**		
BMI at index date (±1 year, the nearest recorded value) (kg/m^2^)	26.6 (26.4 to 26.9)	30.0 (29.8 to 30.1)	1.7 (1.6 to 1.8) for decrease (weight loss) by 5 units	<0.001
Missing	374 (13.9)	3348 (31.8)	-	-
HbA1c at index date (±1 year, the nearest recorded value) (mmol/mol)	64.7 (63.9 to 65.5)	54.7 (54.3 to 55.0)	1.4 (1.3 to 1.4) for increase by 10 units)	<0.001
Missing	231 (8.6)	2,998 (28.5)	-	-
**Subgroup analysis–people without diabetes diagnosis**	***n* = 6,113**	***n* = 24,452**		
BMI at index date (±1 year, the nearest recorded value) (kg/m^2^)	25.0 (24.8 to 25.2)	27.1 (27.0 to 27.2)	1.5 (1.4 to 1.6) for decrease (weight loss) by 5 units	<0.001
Missing	2897 (47.4)	15,833 (64.8)	-	-
HbA1c at index date (±1 year, the nearest recorded value) (mmol/mol)	43.1 (42.6 to 43.6)	38.9 (38.7 to 39.0)	2.1 (1.9 to 2.4) for increase by 10 units)	<0.001
Missing	4152 (67.9)	19,579 (80.1)	-	-

At the time of diagnosis, BMI was lower for cases as compared to controls by nearly 3 BMI units, 25.7 kg/m^2^ (95% CI 25.6 to 25.8) versus 28.4 kg/m^2^ (95% CI 28.3 to 28.5). Crude OR for pancreatic cancer associated with a 5 kg/m^2^ weight loss was 1.6 (95% CI 1.5 to 1.7, *p* < 0.001). Cases were more likely than controls to have elevated HbA1c. The average HbA1c for cases was 55.0 (95% CI 54.4 to 55.7) and for controls it was 48.5 (95% CI 48.2 to 48.7). Crude OR for pancreatic cancer associated with 10 mmol/mol increase in HbA1c was 1.4 (95% CI 1.4 to 1.5, *p* < 0.001). However, at ±1 year of the index date, 3,271 (37%) cases and 19,181 (55%) controls did not have a BMI measurement recorded in their EHRs, and HbA1c was missing for 4,383 (49.9%) cases and 22,577 (64.5%) controls.

### Longitudinal trends in BMI and HbA1c by diabetes status

**Fig 2:**
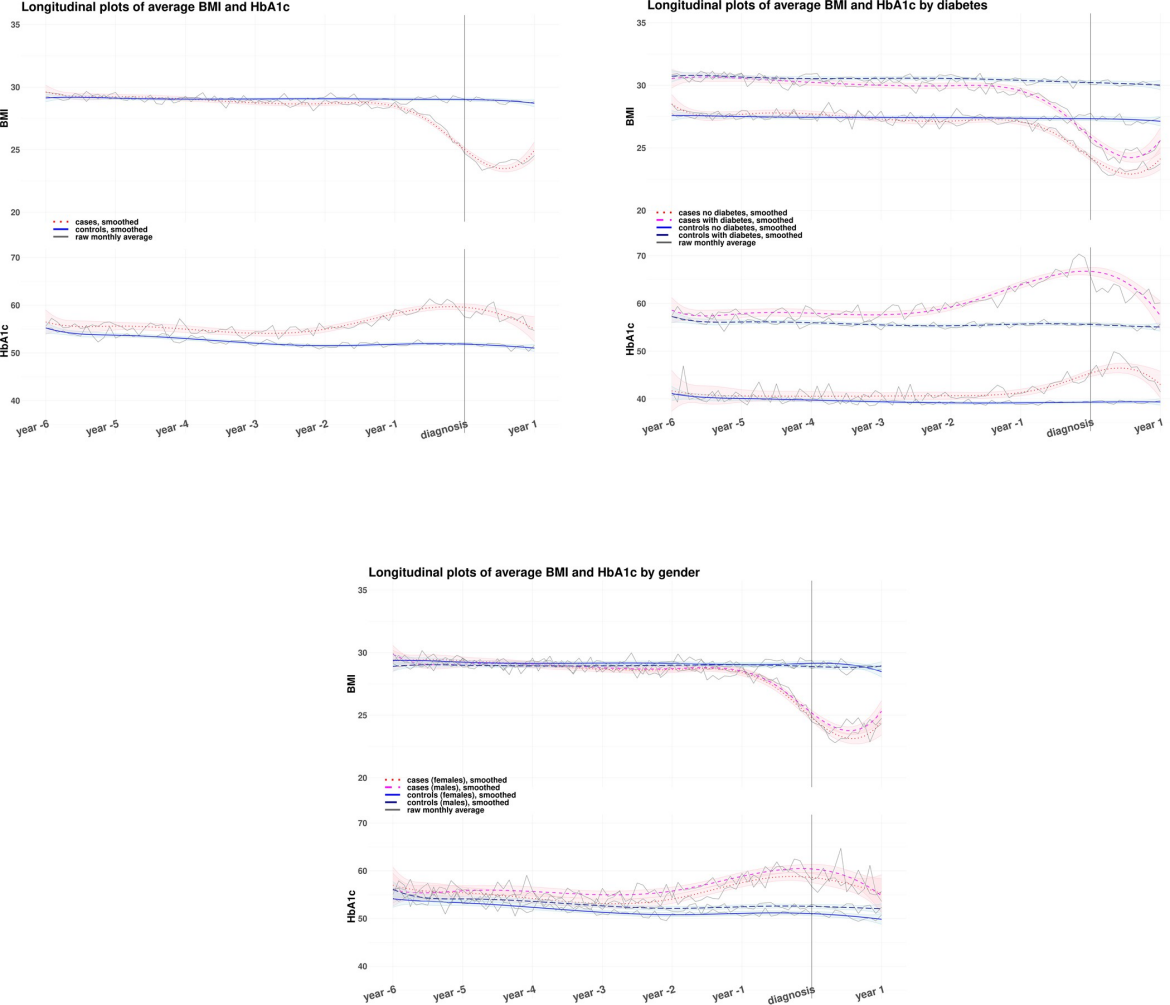
Longitudinal plots, BMI and HbA1c over time from six years before pancreatic cancer diagnosis up to a year after diagnosis. A) total sample, B) and C) subgroup analysis: B) by diabetes status and C) by gender. Raw monthly averages are presented with the grey continuous line. Smoothed trends over time are obtained by fitting changes in BMI and HbA1c as a function of time with linear regression and a three-knot cubic spline to allow non-linearity.

The longitudinal plots in Fig 2 showed weight loss for cases before pancreatic cancer diagnosis which continued after diagnosis.

The weight loss observed for cases started about a year before the diagnosis. However, when looking separately at cases with diabetes and without diabetes (Fig 2B), the drop in BMI for cases with diabetes started about 6 months earlier than for cases without diabetes. By the time they were diagnosed with pancreatic cancer, cases with diabetes lost more weight on average than cases without diabetes (Fig 2B). This is reinforced by the subgroup analysis by diabetes presented in Table 1. We see that at the index date, BMI of cases with diabetes was 3.4 BMI units lower than their matched controls (26.6 [95% CI 26.4 to 26.9] versus 30.0 [95% CI 29.8 to 30.1]). While for cases without diabetes, BMI was 2.1 units lower than that of their matched controls (25.0 [95% CI 24.8 to 25.2] versus 27.1 [95% CI 27.0 to 27.2]). Cases with diabetes who weighed 5 kg/m^2^ less than their matched controls, were 1.7 (1.6 to 1.8, *p* < 0.001) times more likely to be diagnosed with pancreatic cancer, while cases without diabetes who weighed 5 kg/m^2^ less than their matched controls, were 1.5 (1.4 to 1.6, *p* < 0.001) times more likely to be diagnosed with pancreatic cancer. This constitutes a 20% difference in pancreatic cancer risk between people with and without diabetes, if they lose weight. For comparison, there was no difference in weight loss trends between genders and the modelled BMI trends and their 95% CIs presented in Fig 2C overlap.

A similar trend in longitudinal plots was observed for HbA1c as was seen with BMI (Fig 2). Although HbA1c increased before the diagnosis of pancreatic cancer for both subgroups, the change started about 2 years earlier (-3 years versus -1 year) and was larger for cases with diabetes as compared to cases without diabetes. The difference in HbA1c at the index date between cases and controls with diabetes was 10 mmol/mol and between cases and controls without diabetes it was 4.2 mmol/mol. For the subsample with diabetes, the average HbA1c for cases was 64.7 mmol/mol (95% CI 63.9 to 65.5) and for controls it was 54.7 (95% CI 54.3 to 55.0). For the subsample without diabetes the average HbA1c was 43.1 (95% CI 42.6 to 43.6) for cases versus 38.9 (95% CI 38.7 to 39.0) for controls. This is presented in detail in Table 1. The risk of pancreatic cancer associated with an increase in HbA1c was higher for people without diabetes than for people with diabetes. Given a 10 mmol/mol increase in HbA1c, people with diabetes were 1.4 (95% CI 1.3 to 1.4, *p* < 0.001) times more likely to be diagnosed with pancreatic cancer, while people without diabetes were 2.1 (95% CI 1.9 to 2.4, *p* < 0.001) times more likely to be diagnosed with pancreatic cancer than people whose HbA1c did not increase.

### Odds ratios for pancreatic cancer from 5 years before the diagnosis

**Fig 3:**
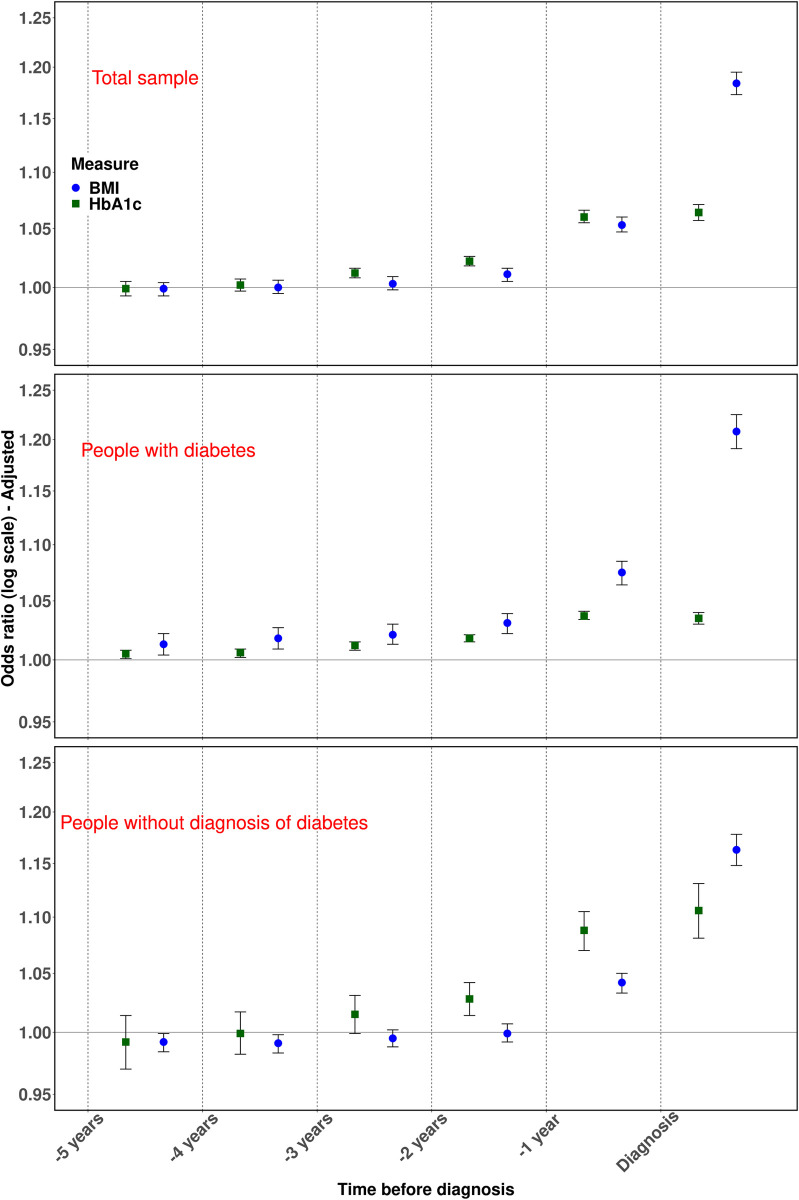
Adjusted odds ratios and 95% confidence intervals for the association of pancreatic cancer with a 1 kg/m2 decrease in body mass index (BMI) between cases and controls (blue circles) and a 1 mmol/mol increase in glycated haemoglobin (HbA1c) (green squares) from 5 years before pancreatic cancer diagnosis. The top plot is for the total study sample. The two further plots show subgroup analysis by the diabetes status.

Cases were more likely than controls to lose weight before the index date (Table 2). This was statistically significant for people with diabetes for at least two years before the pancreatic cancer diagnosis (*p* < 0.001), while for people without diabetes this could only be statistically detected a year before the pancreatic cancer diagnosis (Fig 3). One year before pancreatic cancer diagnosis, the adjusted OR for pancreatic cancer associated with a 1 kg/m^2^ weight loss, was 1.08 (95% CI 1.06 to 1.09, *p* < 0.001) for people with diabetes and 1.04 (95% CI 1.03 to 1.05, *p* < 0.001) for people without diabetes.

**Table 2:** Odds ratios (OR) and 95% confidence intervals (CI) calculated with conditional logistic regression for the association of pancreatic cancer with decrease in body mass index (BMI) i.e., weight loss over time calculated with conditional logistic regression. Subgroup analysis by diabetes. Bolded text indicates statistically significant results. Statistical significance was considered at p < 0.01.

(A) BMI	Cases	Controls	Complete cases analysis				Multiple imputation	
	Mean (95% CI)	Mean (95% CI)	Crude OR (95% CI)	*p*-value	Adjusted OR (95% CI)	*p*-value	Adjusted OR (95% CI)	*p*-value
Total population								
-5 years	28.4 (28.2 to 28.6)	28.6 (28.5 to 28.8)	1.01 (1.00 to 1.02)	0.192	1.01 (0.99 to 1.03)	0.606	1.00 (0.99 to 1.00)	0.614
-4 years	28.5 (28.3 to 28.7)	28.6 (28.5 to 28.8)	1.00 (0.99 to 1.01)	0.671	1.01 (0.99 to 1.02)	0.563	1.00 (1.00 to 1.01)	0.936
-3 years	28.3 (28.1 to 28.5)	28.6 (28.5 to 28.7)	1.01 (1.00 to 1.01)	0.205	1.01 (1.00 to 1.03)	0.111	1.00 (1.00 to 1.01)	0.226
-2 years	28.1 (27.9 to 28.3)	28.7 (28.6 to 28.8)	**1.02 (1.01 to 1.03)**	**<0.001**	**1.03 (1.01 to 1.05)**	**<0.001**	**1.01 (1.01 to 1.02)**	**<0.001**
-1 year	26.6 (26.4 to 26.7)	28.7 (28.6 to 28.8)	**1.07 (1.06 to 1.08)**	**<0.001**	**1.08 (1.06 to 1.09)**	**<0.001**	**1.05 (1.05 to 1.06)**	**<0.001**
Year 0	24.1 (23.9 to 24.3)	28.6 (28.5 to 28.7)	**1.22 (1.19 to 1.24)**	**<0.001**	**1.24 (1.19 to 1.29)**	**<0.001**	**1.18 (1.17 to 1.19)**	**<0.001**
People with diabetes								
-5 years	29.9 (29.6 to 30.2)	30.3 (30.1 to 30.5)	1.01 (0.99 to 1.02)	0.324	1.01 (0.99 to 1.03)	0.511	**1.01 (1.00 to 1.02)**	**0.003**
-4 years	29.8 (29.5 to 30.1)	30.3 (30.1 to 30.5)	1.01 (1.00 to 1.02)	0.263	1.01 (0.99 to 1.03)	0.313	**1.02 (1.01 to 1.03)**	**<0.001**
-3 years	29.8 (29.5 to 30.0)	30.3 (30.1 to 30.5)	1.01 (1.00 to 1.02)	0.101	1.01 (0.99 to 1.03)	0.180	**1.02 (1.01 to 1.03)**	**<0.001**
-2 years	29.3 (29.1 to 29.6)	30.3 (30.1 to 30.4)	**1.03 (1.02 to 1.04)**	**<0.001**	**1.03 (1.02 to 1.05)**	**<0.001**	**1.03 (1.02 to 1.04)**	**<0.001**
-1 year	27.7 (27.5 to 28.0)	30.1 (29.9 to 30.2)	**1.08 (1.06 to 1.09)**	**<0.001**	**1.08 (1.06 to 1.10)**	**<0.001**	**1.08 (1.06 to 1.09)**	**<0.001**
Year 0	24.8 (24.5 to 25.1)	29.9 (29.8 to 30.1)	**1.24 (1.21 to 1.27)**	**<0.001**	**1.24 (1.19 to 1.29)**	**<0.001**	**1.21 (1.19 to 1.23)**	**<0.001**
People without diagnosis of diabetes								
-5 years	27.2 (26.9 to 27.4)	27.3 (27.2 to 27.4)	1.01 (0.99 to 1.02)	0.394	0.85 (0.68 to 1.05)	0.136	0.99 (0.98 to 1.00)	0.022
-4 years	27.3 (27.1 to 27.6)	27.3 (27.1 to 27.4)	1.00 (0.98 to 1.01)	0.466	0.94 (0.84 to 1.04)	0.237	0.99 (0.98 to 1.00)	0.012
-3 years	27.0 (26.8 to 27.3)	27.2 (27.1 to 27.3)	1.00 (0.99 to 1.01)	0.986	1.06 (0.99 to 1.15)	0.101	1.00 (0.99 to 1.00)	0.163
-2 years	26.9 (26.7 to 27.2)	27.3 (27.2 to 27.4)	1.01 (1.00 to 1.02)	0.164	1.01 (0.95 to 1.07)	0.743	1.00 (0.99 to 1.01)	0.892
-1 year	25.6 (25.4 to 25.8)	27.3 (27.1 to 27.4)	**1.06 (1.05 to 1.08)**	**<0.001**	**1.07 (1.02 to 1.12)**	**0.004**	**1.04 (1.03 to 1.05)**	**<0.001**
Year 0	23.6 (23.3 to 23.9)	27.2 (27.0 to 27.3)	**1.18 (1.15 to 1.21)**	**<0.001**	**1.51 (1.16 to 1.97)**	**0.002**	**1.16 (1.15 to 1.18)**	**<0.001**

The increase in HbA1c was statistically significant from three years before pancreatic cancer diagnosis (Table 2). In the year before pancreatic cancer, an increase in HbA1c by 1 mmol/mol between cases and controls, was associated with an adjusted OR of 1.04 (95% CI 1.03 to 1.04, *p* < 0.001) for people with diabetes and 1.09 (95% CI 1.07 to 1.11, *p* < 0.001) for people without diabetes. ORs were getting smaller, and the statistical significance diminished for people with diabetes beyond three years before the diagnosis and for people without diabetes beyond one year before the diagnosis (Fig 3). People continued to lose weight after pancreatic cancer diagnosis with odds ratios increasing from 1.05 (95% CI 1.05 to 1.06, *p* < 0.001) in year -1 to 1.18 (95% CI 1.17 to 1.19, *p* < 0.001) in year 0 (Table 2). However, HbA1c peaked in year -1, and odds ratio remained the same 1.06 (95% CI 1.06 to 1.07, *p* < 0.001) in year -1 and year 0 (Table 3). BMI and HbA1c were more scarcely recorded for people without a diagnosis of diabetes, and this is represented by wider confidence intervals for ORs in the regression models using complete cases analysis.

**Table 3:** Odds ratios (OR) and 95% confidence intervals (CI) for the association of pancreatic cancer with increase in glycated haemoglobin (HbA1c), i.e., hyperglycaemia over time with calculated conditional logistic regression. Subgroup analysis by diabetes. Bolded text indicates statistically significant results. Statistical significance was considered at p < 0.01.

(B) HbA1c	Cases	Controls	Complete case analysis				Multiple imputation	
	Mean (95% CI)	Mean (95% CI)	Crude OR (95% CI)	*p*-value	Adjusted OR (95% CI)	*p*-value	Adjusted OR (95% CI)	*p*-value
Total population								
-5 years	52.4 (51.7 to 53.1)	50.8 (50.4 to 51.2)	1.01 (1.00 to 1.01)	0.021	1.01 (1.00 to 1.02)	0.021	1.00 (0.99 to 1.01)	0.757
-4 years	51.3 (50.6 to 52.0)	50.1 (49.8 to 50.5)	1.01 (1.00 to 1.01)	0.034	1.01 (1.00 to 1.01)	0.183	1.00 (1.00 to 1.01)	0.482
-3 years	**51.4 (50.8 to 52.1)**	**49.4 (49.0 to 49.7)**	**1.01 (1.01 to 1.02)**	**<0.001**	**1.01 (1.01 to 1.02)**	**<0.001**	**1.01 (1.01 to 1.02)**	**<0.001**
-2 years	**52.6 (51.9 to 53.2)**	**49.4 (49.1 to 49.7)**	**1.02 (1.02 to 1.02)**	**<0.001**	**1.03 (1.02 to 1.03)**	**<0.001**	**1.02 (1.02 to 1.03)**	**<0.001**
-1 year	**55.4 (54.8 to 56.1)**	**49.6 (49.3 to 49.8)**	**1.04 (1.04 to 1.05)**	**<0.001**	**1.05 (1.04 to 1.06)**	**<0.001**	**1.06 (1.06 to 1.07)**	**<0.001**
Year 0	**56.5 (55.5 to 57.5)**	**49.3 (49.0 to 49.5)**	**1.03 (1.03 to 1.04)**	**<0.001**	**1.03 (1.02 to 1.05)**	**<0.001**	**1.06 (1.06 to 1.07)**	**<0.001**
People with diabetes								
-5 years	56.1 (55.3 to 57.0)	54.5 (54.1 to 55.0)	1.01 (1.00 to 1.01)	0.033	1.01 (1.00 to 1.02)	0.022	1.01 (1.00 to 1.01)	0.014
-4 years	55.7 (54.9 to 56.5)	54.4 (54.0 to 54.9)	1.01 (1.00 to 1.01)	0.049	1.01 (1.00 to 1.01)	0.192	**1.01 (1.00 to 1.01)**	**0.002**
-3 years	**57.0 (56.2 to 57.8)**	**54.2 (53.8 to 54.6)**	**1.01 (1.01 to 1.02)**	**<0.001**	**1.01 (1.01 to 1.02)**	**<0.001**	**1.01 (1.01 to 1.02)**	**<0.001**
-2 years	**59.2 (58.4 to 60.0)**	**54.5 (54.2 to 54.9)**	**1.02 (1.02 to 1.02)**	**<0.001**	**1.03 (1.02 to 1.03)**	**<0.001**	**1.02 (1.02 to 1.02)**	**<0.001**
-1 year	**65.1 (64.3 to 65.9)**	**54.8 (54.4 to 55.1)**	**1.04 (1.04 to 1.04)**	**<0.001**	**1.05 (1.04 to 1.05)**	**<0.001**	**1.04 (1.03 to 1.04)**	**<0.001**
Year 0	**61.9 (60.7 to 63.1)**	**54.3 (54.0 to 54.7)**	**1.03 (1.02 to 1.03)**	**<0.001**	**1.03 (1.02 to 1.04)**	**<0.001**	**1.04 (1.03 to 1.04)**	**<0.001**
People without diagnosis of diabetes								
-5 years	39.5 (38.9 to 40.2)	39.1 (39.5 to 38.8)	1.05 (0.99 to 1.10)	0.093	0.59 (0.36 to 0.97)	0.039	0.99 (0.97 to 1.01)	0.481
-4 years	39.5 (39.0 to 40.0)	39.2 (38.9 to 39.5)	1.02 (0.99 to 1.06)	0.235	1.00 (0.88 to 1.13)	0.995	1.00 (0.98 to 1.02)	0.935
-3 years	39.8 (39.2 to 40.3)	39.0 (38.7 to 39.2)	1.01 (0.99 to 1.02)	0.474	1.03 (0.96 to 1.10)	0.478	1.02 (1.00 to 1.03)	0.062
-2 years	**40.2 (39.8 to 40.7)**	**38.9 (38.7 to 39.2)**	1.02 (1.00 to 1.04)	0.028	1.04 (0.97 to 1.10)	0.269	**1.03 (1.01 to 1.04)**	**<0.001**
-1 year	**42.6 (42.1 to 43.1)**	**38.9 (38.7 to 39.1)**	**1.09 (1.07 to 1.10)**	**<0.001**	**1.09 (1.04 to 1.14)**	**<0.001**	**1.09 (1.07 to 1.11)**	**<0.001**
Year 0	**45.8 (44.4 to 47.2)**	**39.1 (38.9 to 39.3)**	**1.07 (1.04 to 1.10)**	**0.001**	1.24 (1.04 to 1.47)	0.015	**1.10 (1.08 to 1.13)**	**<0.001**

## Discussion

### Summary

This is the first population-based study, using a large, nationally representative database in the UK to investigate the association of weight loss and rising HbA1c with pancreatic cancer over time. We evaluated temporal patterns in ORs for the association of BMI and HbA1c with pancreatic cancer in six separate timepoints from 5 years before pancreatic cancer diagnosis. This contributes to the evidence on how early the association can be useful in cancer detection. The study demonstrated that the association of weight loss with pancreatic cancer was statistically significant from two years before pancreatic cancer diagnosis, and the association of rising HbA1c was statistically significant from three years before., It also showed that weight loss in people with diabetes was associated with higher risk than in people without diabetes, and that hyperglycaemia in people without diabetes was associated with higher risk than in people with diabetes.

### Strengths and limitations

This study has several strengths. Firstly, it uses a large, nationally representative (England) database [17]. Secondly, because the included primary care practices are a part of a research network and they are vetted for quality, the quality of the data is high [20]. Although there are missing data and some errors in coding can occur (for example the erroneous coding of the type of diabetes observed in this study), these are unlikely to influence the results of this study. Thirdly, this study used routinely collected data which is representative of real-life as seen in clinical practice. Therefore, any algorithms developed using the evidence presented in this study, will have clinical applications.

The matched case-control design is a strength. It limited the effect of age, gender, and diabetes on other covariates. We undertook subgroup analysis according to the diabetes status. This is because, not everyone with pancreatic cancer will develop diabetes and the prediction algorithms that focus on people with new diabetes leave out a significant proportion of cases. The stratified analyses are important to improve understanding of the predictive utility in different groups of patients.

BMI and HbA1c are simple measures, routinely collected in clinical practice. However, the challenges were the irregular testing and missing data. This is an inherent characteristic of routine data, not unique to this database, and other primary care databases would be similarly affected. We applied recommended and previously proven statistical approaches to overcome this [24, 25]. In addition, BMI and HbA1c were more scarcely recorded for people without a diagnosis of diabetes than for people with diabetes, and this resulted in wider confidence intervals for ORs in the regression models using complete case analysis.

### Findings in the context of previous research

Depending on the location within the organ, pancreatic cancer can cause endo- and exocrine deficiency of the pancreas leading to hyperglycaemia, diabetes, weight loss and/or malnutrition [26]. Given that the majority of patients (80% to 85%) experience hyperglycaemia one to three years before pancreatic cancer diagnosis [27, 28], and 70% to 75% experience weight loss starting about a year prior to diagnosis [6, 29–31], this makes these metabolic changes important candidates for cancer markers. The advantage of using HbA1c and BMI is that in many people hyperglycaemia and weight loss happen years before pancreatic cancer-specific symptoms such as jaundice. However, the challenge is that hyperglycaemia and weight loss are non-specific to pancreatic cancer. HbA1c increases with age and about one-third of US adults have prediabetes and 27% of people aged 65 and over have diabetes [32]. With diabetes being so much more prevalent than pancreatic cancer, it is difficult in clinical practice to recognise pancreatic cancer-induced hyperglycaemia. In addition, weight loss in people with newly diagnosed diabetes is often a recommended management strategy, and some diabetes medications can induce weight loss. Moreover, hyperglycaemia and diabetes themselves can lead to unexpected weight loss.

Diabetes is a known risk factor and, a large proportion of prediction algorithms published to date focuses on people with newly developed diabetes. This approach could be challenged because diabetes itself is a highly undiagnosed disease. It is estimated that around 3% of US adults have undiagnosed diabetes and that more than 20% of all diabetes cases go undiagnosed [32]. This is reinforced by the results of this study. The estimated prevalence of diabetes in people with pancreatic cancer is around 40% to 50% [14, 15], but we recorded that only around 30% received a diabetes diagnosis. In addition, diabetes was diagnosed in the same year or after pancreatic cancer for 59% of cases with type 1 and 26% of cases with type 2 diabetes. It is therefore possible that for many people, diabetes indicated a progression to an advanced stage of cancer. However, this study showed that cases without diabetes experienced hyperglycaemia which was linked to an increased risk of pancreatic cancer from two years before the diagnosis.

### Clinical implications and future research

In the context of the ageing population and multimorbidity, the issues involved in using HbA1c and BMI in early detection are complex. Data-driven risk prediction algorithms can serve as important tools that incorporate a carefully selected combination of symptoms and risk factors to stratify patients. These tools can be applied to routine data to identify patients at risk. However, this relies on the quality and completeness of data. Regular HbA1c and BMI measurements in primary care would not only improve pre-diabetes and diabetes diagnosis, but also improve early pancreatic cancer diagnosis. They would also improve the quality of routine data for research.

This evidence in this study could be used in clinical practice to aid pancreatic cancer diagnosis earlier than is currently achieved. However, more research is needed to tailor its use for specific groups of patients who experience weight loss and hyperglycaemia in early stages of cancer. Our findings align with research published to date. In a case-control study that investigated glucose levels, Sharma et al. 2018 also found that the mean duration of hyperglycaemia was 30 to 36 months before pancreatic cancer diagnosis [28]. This indicates an important window of opportunity for earlier diagnosis that could make a significant improvement in survival. At present, the majority (80% to 85%) of patients are diagnosed with a locally advanced or metastatic disease when the curative surgery option is no longer available [28, 33]. A study by Pelaez-Luna et al. suggested that detection even 6 months earlier could improve chances for a diagnosis of a resectable disease [34].

Future work is planned using the dataset presented in this study. This will be to evaluate the predictive value of BMI and HbA1c when used in combination with each other and other symptoms and risk factors. In the first instance, we will evaluate the specificity and sensitivity of the ENDPAC algorithm [10, 11]. This will then be implemented in a prospective study to audit primary care records, identify patients at a high risk of pancreatic cancer and recommend them for onwards investigations including blood markers such as CA19-9 and an abdominal scan. However, before such a trial with patients could be undertaken, more testing of the predictive algorithms for routine data is needed to avoid the unnecessary burden of false positives. This study is an important first step towards evaluating prediction algorithms from routine data and future trials with patients which could improve practice.

## Conclusions

Weight loss and rising HbA1c could be used in clinical practice to aid pancreatic cancer diagnosis earlier than is currently achieved. However, because they are both nonspecific to pancreatic cancer and vary according to the diabetes status, they should be used together, and in combination with other symptoms and risk factors to improve early diagnosis. Data-driven algorithms could facilitate this for clinicians. High quality routine data and regular BMI and HbA1c measurements are required to facilitate future research and implementation in clinical practice.

### Ethical issues and approvals

Dataset was pseudonymised and researchers did not have access to any patient-identifiable information. All the processing and analysis of data were conducted within the ORCHID secure network. Participant consent was on an opt-out basis. Data were excluded for individuals who opted out of their medical records being used for research. Written consent was not feasible because we do not hold patient identifiers and cannot contact individual patients. The study protocol and data request were approved by the RCGP and University of Oxford Joint Research and Surveillance Committee (approval number RSC_0420). The ethical review was conducted the University of Surrey using the Self-Assessment for Governance and Ethics tool (review number 514292-514283-55148034) and the Health Research Authority (HRA) Medical Research Council (MRC) decision tool: http://hra-decisiontools.org.uk/ethics and concluded that further ethical approval was not required.
